# Association between periodontitis and disc structural failure in older adults with lumbar degenerative disorders: A prospective cohort study

**DOI:** 10.1186/s12893-023-01950-7

**Published:** 2023-03-18

**Authors:** Xiaolong Chen, Dong Xue, Ying Zhao, Peng Cui, Peng Wang, Yu Wang, Shi-bao Lu

**Affiliations:** 1 Department of Orthopaedics, Xuanwu Hospital Capital Medical University, 100053 Beijing, China; 2 Department of Stomatology, Xuanwu Hospital Capital Medical University, 100053 Beijing, China

**Keywords:** Periodontitis, Intervertebral disc degeneration, Endplate change, Older adult, Lumbar degenerative disorders

## Abstract

**Background:**

Bacterial microbiome as a putative trigger of inflammation might indicate the cascade of mouth-gut-disc axis for causing intervertebral disc (IVD) structural failures (such as IVD degeneration and endplate change) processed. However, direct evidence for the mouth-gut-disc axis still unclear. Therefore, it is interesting to explore periodontal inflammation related to IVD structural failures and clinical outcomes.

**Methods:**

This prospective cohort study enrolled older adults (aged ≥ 75 years) who scheduled to undergo elective open lumbar spine surgery. Demographic, radiological, clinical, and periodontal parameters were recorded. Independent samples t-test and Pearson’s correlation analysis were calculated.

**Results:**

A total of 141 patients with lumbar degenerative disorders (56 males and 85 females; age 79.73 ± 3.34 years) were divided into edentulous group (19 patients), No/Mild group (84 patients), and Moderate/Severe group (38 patients). The incidence rates of IVD degeneration in each lumbar segmental level based on Pfirrmann grade and endplate change in the fourth and fifth lumbar vertebrae, and Visual Analogue Scale (VAS) low back pain (LBP) and leg pain of patients at preoperative in dentate group was significantly higher compared with edentulous group, especially the comparisons between Moderate/Severe and edentulous groups. There were no significant differences in the range of motion, lumbar lordosis, pelvic incidence, pelvic tilt, sacral slope, and disc height between dentate and edentulous groups. There was a positive association between plaque index (PLI) and pain scores (VAS LBP: r = 0.215, P = 0.030 and VAS leg pain: r = 0.309, P = 0.005), but no significant difference in Oswestry disability index (ODI) score.

**Conclusion:**

Results show that the severity of periodontitis is associated with higher incidence rates of IVD degeneration and endplate change and clinical outcomes in older adults with lumbar degenerative disorders. Furthermore, the discovery of these relationships unveils a novel mechanism through which the alterations in oral microbiome composition potentially promote IVD degeneration and pain.

## Introduction

China is experiencing the fastest aging of its population, with up to 200 million people older than 65, accounting for 13.5% of the total China population [1]. With the improvement of medical standards and life expectancy, individuals 75 years or older represent the fastest-growing segment of people, with increasing incidence and prevalence rates of lumbar degenerative disorders [2, 3]. Low back pain (LBP) is the main feature of lumbar degenerative disorders which is the most significant cause of disability and lost productivity worldwide. Within the vast differential of LBP, the most common source is intervertebral disc (IVD) degeneration. Radiographic changes supportive of the degeneration of IVD are narrowing of the disc space and/or articular facets, narrowing and/or increased opacity of the intervertebral foramen, endplate change, presence of mineralized disc material within the vertebral canal and vacuum phenomenon [4, 5]. In theory, the microstructure change of IVD could be reflected in the change in disc height (DH). The change in IVD height influences the load-carrying capacity of the spinal column, and morphologic abnormalities such as IVD space narrowing and thinning which is potentially associated with acute or chronic disabilities of the lumbar spine [6]. Pfirrmann et al. [7] developed a scoring system to evaluate disc degeneration based on the changes in DH in MRI, and the relationship between quantitative measures of DH with the Pfirrmann disc degeneration scoring system has been investigated [8]. In addition, most patients with endplate changes also have concurrent evidence of disc degeneration [9].

Numerous factors may trigger the degenerative process. In the aging IVD, structural defects or failures such as neovascularization, tears, radial fissures, endplate change, and herniation become more common. Excessive mechanical loading leads to the structural changes of IVD and a cascade of cell-mediated responses, causing further disruption, which has been referred to as the causality of IVD degeneration. Aging, genetic factors, mechanical loading, inadequate metabolite transport, smoking, natural history, and obesity have been considered as the potential factors for triggering IVD degenerative process. Inflammation is the major concern around IVD degeneration [10], however, the exact cause and nature of such inflammation are still unclear. A recent review summarizes the gut-disc axis (e.g., microbiome dysbiosis in the gastrointestinal system and mouth) possible influence on IVD degeneration and LBP [11]. The evidence for supporting the hypothesis is required.

Many microbial communities have been harbored in the subgingival crevice. Shifts in the composition of these communities occur with the development of gingivitis and periodontitis, which is regarded as one of the most likely causes of periodontal health deterioration [12]. The association between the oral microbiome (the taxonomic composition of the microbiome) and periodontal diseases has been reported [13]. Moreover, the oral microbiota promotes systemic inflammation which is suggested to be the link between periodontal disease and the development of metabolic syndrome [14, 15]. Further, Propionibacterium acnes (P. acnes) is considered one of the responsible for inflammation of the gingivitis, consequently leading to various diseases in the skin, airways, eyes, heart valves, joints, and others [16]. The infection of IVD by *P. acnes* has been reported as the putative trigger of inflammation which might play a key role in the IVD degeneration process [17]. Taken together, these factors might indicate the cascade of the gut-disc axis in the IVD degeneration process: microbiome dysbiosis in the mouth causes periodontal inflammation and precipitates a cascade of systematic inflammation, leading to further IVD degeneration. However, the direct evidence for the gut-disc axis is still unclear. Therefore, it is interesting to explore the periodontal inflammation is related to the severity of IVD degeneration and endplate change.

To further our understanding of the affection of gut-disc axis on the IVD degeneration and endplate change, may serve to improve regulation and management of LBP in patients with IVD degeneration in older adults. The aims of this study were: (1) to investigate the periodontal inflammation in relation to IVD degeneration and endplate change, and (2) to evaluate the relationship between periodontal inflammation and clinical outcomes.

## Materials and methods

### Study design

This single-center prospective non-randomised study as one part of a previously registered study was registered Chinese clinical trial registry (ChiCTR1800020363) on 25th December 2018 and approved by the Medical Research Ethics Committee of Xuanwu Hospital of Capital Medical University. All participants had consented to demographic data, radiological data, and clinical scores to be used for research.

### Participants

Between July 2019 and December 2021, 141 older adults (aged ≥ 75 years) who were scheduled to undergo elective open lumbar spine surgery and have a periodic oral evaluation before surgery at Xuanwu Hospital Capital Medical University were enrolled.

Participants met the following inclusion criteria: [1] aged ≥ 75 years old; and [2] on the waiting list for undergoing elective open lumbar spine surgery.

Exclusion criteria were: [1] history of spinal deformity, tumor, infection, and trauma in the region of the lumbar spine; [2] history of lumbar spine surgery; [3] severe organic disease, systemic metabolic disease, and systematic disease; [4] participants were recommended to conservative treatment or minimally invasive surgery; and [5] participants declined to the project.

Participants were assessed during the admission period by surgeons.

### Demographic data

Demographic data of the patient’s age, gender, body mass index (BMI), duration of symptoms, and diagnosis of lumbar degenerative diseases were collected.

### Periodontal parameters

Due to the older adults (aged ≥ 75 years) enrolled in this project, all the participants were divided into dentate group and edentulous group. The number of remaining teeth, the history of periodontal treatment (yes or no), periodontal maintenance (yes or no), the history of mouthwash (yes or no), the common causes of missing teeth (e.g., periodontal disease, cavities, injury, etc.), plaque index (PLI), bleeding index (BI), the number of loose teeth, gingival recession (GR, measured from the cementoenamel junction to the gingival margin), and probing depth (PD, measured from the gingival margin to the bottom of the pocket in six sites including mesiobuccal, buccal, distobuccal, mesiolingual, lingual, and distolingual, except the third molars) were recorded [18, 19].

According to the diagnosis and classification criteria of periodontitis from the Center for Disease Control and Prevention in partnership with the American Academy of Periodontology (CDC-PAAP) [20], periodontitis was classified into mild, moderate, and severe. Mild periodontitis was defined by the presence of periodontal pockets in ≥ 2 interproximal sites with a clinical attachment level of ≥ 3 mm, in ≥ 2 interproximal sites with a PD ≥ 4 mm (for different teeth), or in one site with a PD ≥ 5 mm. Clinical attachment levels (CAL) were only measured when a periodontal pocket ≥ 3 mm was defined as the algebraic sum of PD and GR. Moderate periodontitis was referred to as ≥ 2 interproximal sites with ≥ 4 mm CAL (not on the same tooth) or ≥ 2 interproximal sites with PD ≥ 5 mm, also not on the same tooth. Severe periodontitis was referred to as having ≥ 2 interproximal sites with CAL ≥ 6 mm (not on the same tooth) and ≥ 1 interproximal site(s) with PD ≥ 5 mm. Based on the classification, all the participants were divided into three groups: No/Mild periodontitis group, Moderate/Severe periodontitis group, and edentulous group.

### Clinical assessment

After obtaining written consent, the participants were asked to complete two questionnaires during the admission period. The questionnaires are: (1) Visual Analogue Scale (VAS; 0 - no pain; 10 - worst pain imaginable) of LBP and leg pain, and (2) Oswestry disability index (ODI; a validated tool for assessing function and disability on 10 items, each item was manually rated with 5 points for six possible responses (the first statement is marked the section score = 0; the last statement is marked the section score = 5), giving a potential score between 0 and 100%.

### Radiological assessment

All participants’ MR images were obtained with a 3.0 T Trio Tim scanner (Siemens, Erlangen, Germany). Sagittal T2-weighted fast spin-echo (FSE), sagittal T1-weight FSE, and axial T2-weighted scans were performed. Field of view (FOV), repetition time (TR) / echo time (TE), matrix size, slice thickness, slice per slab, and the number of excitations (NEX) are 310 * 310 mm, 550 ms / 9.6 ms, 320 * 320, 4.0 mm, 11, and 2 during the sagittal T1-weighted scan, respectively. The FOV, the TR/TE, matrix size, slice thickness, slice per slab, and NEX are 310 * 310 mm, 2700 ms / 97 ms, 320 * 320, 4.0 mm, 11, and 2 during the sagittal T2-weighted scan, respectively. The FOV, the TR/TE, matrix size, slice thickness, slice per slab, and NEX are 210 * 210 mm, 3400 ms / 102 ms, 320 * 320, 4.0 mm, 15, and 2 during the axial T2-weighted scan, respectively. The mid-sagittal section of the T2-weighted slice was selected by the research team for measuring DH. Apple MacBook with integrated touchpads and the Philips DICOM Viewer (Philips, Best, the Netherlands) was used to measure the DH for reducing the potential bias in the measurements.

The Pfirrmann score is used to evaluate IVD degeneration based on the distinction of the nucleus pulpous and the annulus fibrosis, signal intensity of the IVD, and height of IVDs (the mean of the sum of the anterior, middle, and posterior IVD height was referred as the height of IVDs) [7]. Pfirrmann grade ≥ III is defined as disc degeneration [21], which was used to allocate the participants into IVD degeneration group (+, Pfirrmann grade ≥ III) and non-degeneration group (-, Pfirrmann grade < III). Due to the older adults who were scheduled to undergo elective open lumbar spine surgery with severe IVD degeneration, the participants were allocated into moderate IVD degeneration (Pfirrmann grade IV) and severe IVD degeneration (Pfirrmann grade V).

Modic changes summarized and classified the signal intensity changes of vertebral endplates and subchondral bone into three types by using magnetic resonance imaging (MRI) [22, 23]. Based on Modic classification, participants were allocated into non-endplate change (type I) and endplate change (type II and III).

Anterior, middle, and posterior IVD height of each segmental level, range of motion (ROM) of each segmental level, lumbar lordosis (LL), pelvic incidence (PI), pelvic tilt (PT), and sacral slope (SS) were measured on the X-ray (e.g., lateral, flexion, and extension).

### Statistical analysis

The continuous data are presented as mean ± standard deviation (SD). The dichotomous data are presented as numbers and percentages. Comparison of age, BMI, duration of pain, and clinical outcomes (VAS LBP, VAS leg pain, and ODI) between the groups were made by the one-way analysis of variance. The independent samples t-test was used to compare the clinical periodontal parameters PLI, BI, GR, PD, tooth count, clinical outcomes (VAS LBP, VAS leg pain, and ODI), and continuous radiological parameters (disc height, LL, PI, PT, SS, and ROM) between the edentulous group and dentate group. Subgroup analysis based on periodontal disease severity was performed (No/Mild group versus Moderate/Severe group). The IVD degeneration as well as endplate change was compared between groups by the Χ^2^ test. The normality of variables has been evaluated. Pearson’s correlation analysis was calculated as a measure of associations between periodontal parameters and radiological data and clinical outcomes. Correlations less than 0.3, between 0.3 and 0.5, between 0.5 and 0.7, and greater than 0.7 are indicative of weak, moderate, strong, and very strong.

Two researchers (PC and PW) conducted the measurements. Intra- and inter-rater reliability was evaluated with intra-class correlation coefficient (ICC) and their 95% confidence intervals (95% CI). Values of ICC less than 0.5, between 0.5 and 0.75, between 0.75 and 0.9, and greater than 0.90 are indicative of poor, moderate, good, and excellent reliability, respectively [24]. SPSS v24.0 (SPSS Inc., Chicago, IL., USA) was used for the statistical analysis. A P value less than 0.05 is statistically significant.

## Results

### Patient characteristics

A total of 141 patients with lumbar degenerative disorders (56 males and 85 females; the age of 79.73 ± 3.34 years, ranging from 75 to 90 years; BMI 24.62 ± 4.30 kg/m^2^) were enrolled. All 141 patients were divided into three groups (19 patients in the edentulous group, 84 patients in the No/Mild group, and 38 patients in the Moderate/Severe group) according to the presence of teeth and periodontal disease severity. Demographic data, periodontal data, radiological data, and clinical data are presented in Table 1. The mean duration of pain was 77.56 weeks (range from 462 to 18 weeks). Of these, 84 (596%) patients were diagnosed with lumbar spinal stenosis, 37 (26.2%) patients with lumbar disc herniation, 11 (7.8%) patients with scoliosis, and 9 (6.4%) patients with lumbar spondylolisthesis Disc degeneration and non-degeneration were diagnosed in 36 and 24 patients by using Pfirrmann grade, respectively. Low fat infiltration was diagnosed in 29 patients. As shown in Table 1, the mean VAS LBP, VAS leg pain, and ODI scores were 7.82 ± 1.81, 7.42 ± 1.89, and 26.64 ± 9.90 at preoperative, respectively.

**Table 1:** Demographic data, periodontal parameters, and clinical outcomes between dentate and edentulous groups

	Dentate group	Edentulous group	Total	P value
**Number of patients**	122	19	141	-
**Female, n (%)**	72 (59)	13 (68.4)	85 (60.3)	0.436
**Age (years)**	79.63 ± 3.45	80.37 ± 2.48	79.73 ± 3.34	0.208
**BMI (kg/m** ^**2**^ **)**	24.90 ± 4.37	22.83 ± 3.42	24.62 ± 4.30	0.056
**Diagnosis, n (%)**				0.001^***^
**Lumbar spinal stenosis**	77 (63.1)	7 (36.8)	84 (59.6)	
**Lumbar spondylolisthesis**	6 (4.9)	3 (15.8)	9 (6.4)	
**Lumbar disc herniation**	29 (23.8)	8 (42.1)	37 (26.2)	
**Scoliosis**	10 (8.2)	1 (5.3)	11 (7.8)	
**Number of remaining teeth**	19.08 ± 7.30	0	19.48 ± 6.90	0.000^***^
**History of periodontal treatment (yes), n (%)**	9 (7.4)	0	9 (6.4)	
**Periodontal maintenance (yes), n (%)**	7 (5.7)	0	7 (5.0)	
**History of mouthwash (yes), n (%)**	2 (1.6)	0	2 (1.4)	
**Common cause of missing teeth, n (%)**				0.003^***^
**Periodontal disease**	30 (24.6)	13 (68.4)	43 (30.5)	
**Cavities**	76 (62.3)	4 (21.1)	80 (56.7)	
**Injury**	16 (13.1)	2 (10.5)	18 (12.8)	
**Plaque index (PLI)**	2.01 ± 0.72	0	2.00 ± 0.72	0.000^***^
**Bleeding index (BI)**	1.41 ± 0.97	0	1.41 ± 0.97	
**Gingival recession (GR)**	1.65 ± 0.93	0	1.66 ± 0.92	
**Probing depth (PD)**	2.59 ± 0.78	0	2.61 ± 0.75	
**VAS LBP**	4.82 ± 6.32	2.81 ± 2.27	3.61 ± 1.96	0.041^*^
**VAS leg pain**	4.41 ± 2.78	2.82 ± 2.75	4.22 ± 2.81	0.045^*^
**ODI**	46.19 ± 17.94	51.46 ± 18.77	44.81 ± 20.23	0.644
**Duration of pain (weeks)**	55.73 ± 17.78	52.77 ± 14.77	54.56 ± 16.57	0.567
**Pfirrmann grade of disc degeneration**				
**L1-L2, n (%)**				0.039^*^
**No degeneration**	30 (24.6)	9 (47.4)	40 (28.4)	
**Degeneration**	92 (75.4)	10 (52.6)	101 (71.6)	
**L2-L3, n (%)**				0.030^*^
**No degeneration**	24 (19.6)	8 (42.1)	32 (22.7)	
**Degeneration**	98 (80.4)	11 (57.9)	109 (77.3)	
**L3-L4, n (%)**				0.030^*^
**No degeneration**	24 (19.6)	8 (42.1)	32 (22.7)	
**Degeneration**	98 (80.4)	11 (57.9)	109 (77.3)	
**L4-L5, n (%)**				0.008^**^
**No degeneration**	12 (9.8)	6 (31.6)	18 (12.8)	
**Degeneration**	110 (90.2)	13 (68.4)	123 (87.2)	
**L5S1, n (%)**				0.019^*^
**No degeneration**	14 (11.5)	6 (31.6)	20 (14.2)	
**Degeneration**	108 (88.5)	13 (68.4)	121 (85.8)	
**Endplate change**				
**L1 (yes), n (%)**	2 (1.6)	1 (5.3)	3 (3.6)	0.354
**L2 (yes), n (%)**	13 (10.7)	1 (5.3)	14 (16.7)	0.693
**L3 (yes), n (%)**	18 (14.8)	2 (10.5)	20 (23.8)	1.000
**L4 (yes), n (%)**	35 (28.7)	1 (5.3)	36 (25.5)	0.044^*^
**L5 (yes), n (%)**	35 (28.7)	1 (5.3)	36 (25.5)	0.044^*^
**S1 (yes), n (%)**	19 (15.6)	2 (10.5)	18 (12.8)	0.739
**ROM (°)**				
**L1-L2**	2.775 ± 1.98	3.01 ± 1.98	2.79 ± 1.97	0.618
**L2-L3**	3.42 ± 2.29	3.18 ± 2.05	3.38 ± 2.25	0.683
**L3-L4**	3.97 ± 2.69	3.41 ± 2.58	3.89 ± 2.67	0.413
**L4-L5**	4.50 ± 3.01	5.03 ± 3.15	4.57 ± 3.02	0.493
**L5S1**	4.89 ± 2.58	5.49 ± 3.31	4.97 ± 2.69	0.383
**LL**	31.19 ± 14.00	31.23 ± 11.00	31.20 ± 13.60	0.990
**PI**	51.70 ± 10.89	50.68 ± 11.06	51.56 ± 10.88	0.707
**PT**	24.48 ± 9.46	27.37 ± 11.94	24.87 ± 9.83	0.234
**SS**	27.25 ± 8.40	23.29 ± 8.94	26.72 ± 8.55	0.060
**Disc height (mm)**				
**L1-L2**				
**Anterior**	9.75 ± 2.49	10.23 ± 2.47	9.82 ± 2.48	0.438
**Middle**	9.65 ± 2.45	10.20 ± 2.26	9.73 ± 2.43	0.362
**Posterior**	6.78 ± 1.67	6.61 ± 1.88	6.75 ± 1.69	0.685
**L2-L3**				
**Anterior**	10.773 ± 3.12	10.81 ± 2.66	10.74 ± 3.05	0.919
**Middle**	10.10 ± 2.84	9.82 ± 2.70	10.06 ± 2.81	0.691
**Posterior**	7.18 ± 1.95	7.53 ± 2.30	7.23 ± 1.99	0.467
**L3-L4**				
**Anterior**	12.40 ± 3.90	12.13 ± 3.56	12.36 ± 3.85	0.778
**Middle**	10.87 ± 3.30	10.85 ± 3.11	10.87 ± 3.26	0.984
**Posterior**	7.87 ± 2.24	8.43 ± 2.02	7.95 ± 2.21	0.309
**L4-L5**				
**Anterior**	12.66 ± 3.82	12.16 ± 2.88	12.59 ± 3.70	0.588
**Middle**	10.84 ± 3.33	10.46 ± 2.45	10.79 ± 3.22	0.637
**Posterior**	8.51 ± 2.42	8.29 ± 1.85	8.48 ± 2.35	0.712
**L5S1**				
**Anterior**	14.01 ± 4.09	14.57 ± 4.44	14.08 ± 4.12	0.583
**Middle**	10.74 ± 3.60	11.77 ± 3.17	10.88 ± 3.55	0.239
**Posterior**	8.48 ± 2.30	8.68 ± 1.49	8.51 ± 2.20	0.705

BMI - body mass index, VAS - visual analogue scale, LBP - low back pain, ODI – Oswestry disability index, LL - lumbar lordosis; PI - pelvic incidence; PT - pelvic tilt; SS - sacral slope, ROM – range of motion; Continuous and dichotomous data are presented as mean ± standard deviation and number (percentage)

Significant difference ^*^ P < 0.05, ^**^ P < 0.01, ^***^ P < 0.001

### Comparison of demographic, Periodontal, Radiological, and Clinical Data between Dentate and Edentulous Groups

The diagnosis of included patients was statistically significant, but no significant difference in age, gender, and BMI. Periodontal clinical parameters (including the number of remaining teeth, history of periodontal treatment, history of mouthwash, a common cause of missing teeth, PLI, BI, GR, and PD were significantly different between dentate and edentulous groups (Table 1).

The VAS LBP (P = 0.041) and VAS leg pain (P = 0.045) of patients preoperatively in the dentate group were significantly higher compared with the edentulous group. There were no significant differences between dentate and edentulous groups regarding ODI score in disability (Table 1).

The incidence rate of IVD degeneration in each lumbar segmental level based on Pfirrmann grade (L1-L2: P = 0.039, L2-L3: P = 0.030, L3-L4: P = 0.030, L4-L5: P = 0.008, L5S1: P = 0.019) and endplate changes in the fourth and fifth lumbar vertebrae (L4: P = 0.044, L5: P = 0.044) in the dentate group were significantly higher compared with the edentulous group. There were no significant differences in endplate changes in the first, second, third lumbar vertebrae and sacrum, ROM of each lumbar segmental level, LL, PI, PT, SS, and disc height of each lumbar segmental level between dentate and edentulous groups (Table 1).

### Comparison of demographic, Periodontal, Radiological, and Clinical Data between No/Mild, Moderate/Severe, and Edentulous Groups

#### No/Mild group versus Moderate/Severe group

Periodontal clinical parameters PLI, BI, GR, and PD were significantly different between No/Mild and Moderate/Severe groups according to the periodontal disease severity (P = 0.000, Table 2). There were no significant differences between dentate groups regarding demographic, radiological, and other periodontal data (including the number of remaining teeth, history of periodontal treatment, history of mouthwash, and a common cause of missing teeth) (Tables 2 and 3).

**Table 2:** Demographic data, periodontal parameters, and clinical outcomes of included patients

	No/Mild group	Moderate/Severe group	Edentulous group
**Number of patients**	65	57	19
**Female, n (%)**	42 (64.6)	19 (50)	13 (68.4)
**Age (years)**	79.39 ± 3.42	80.16 ± 3.42	80.37 ± 2.48
**BMI (kg/m** ^**2**^ **)**	24.73 ± 4.57	24.51 ± 3.87	22.83 ± 3.42
**Diagnosis, n (%)**			
**Lumbar spinal stenosis**	43 (66.1)	34 (59.7)	7 (36.8)
**Lumbar spondylolisthesis**	2 (3.1)	4 (7.0)	3 (15.8)
**Lumbar disc herniation**	18 (27.7)	11 (19.3)	8 (42.1)
**Scoliosis**	2 (3.1)	8 (14.0)	1 (5.3)
**Number of remaining teeth**	19.63 ± 7.38	18.96 ± 6.96	0
**History of periodontal treatment (yes), n (%)**	6 (9.2)	3 (5.3)	0
**Periodontal maintenance (yes), n (%)**	4 (6.2)	3 (5.3)	0
**History of mouthwash (yes), n (%)**	1 (1.5)	1 (1.8)	0
**Common cause of missing teeth, n (%)**			
**Periodontal disease**	8 (12.3)	22 (38.6)	13 (68.4)
**Cavities**	48 (73.9)	28 (49.1)	4 (21.1)
**Injury**	9 (13.8)	7 (12.3)	2 (10.5)
**Plaque index (PLI)**	1.79 ± 0.70	2.27 ± 0.67 ^a***^	-
**Bleeding index (BI)**	0.95 ± 0.81	1.99 ± 0.89 ^a***^	
**Gingival recession (GR)**	1.35 ± 0.81	2.02 ± 0.93 ^a***^	
**Probing depth (PD)**	2.19 ± 0.40	3.12 ± 0.81 ^a***^	
**VAS LBP**	4.43 ± 2.08 ^b*^	4.02 ± 2.02 ^c*^	2.81 ± 2.27
**VAS leg pain**	4.78 ± 2.32 ^b**^	3.63 ± 2.41 ^c*^	2.82 ± 2.75
**ODI**	48.67 ± 17.29	43.21 ± 18.88	51.46 ± 18.77
**Duration of pain (weeks)**	56.42 ± 16.81	53.33 ± 16.24	52.77 ± 14.77

BMI - body mass index, VAS - visual analogue scale, LBP - low back pain, ODI – Oswestry disability index; Continuous and dichotomous data are presented as mean ± standard deviation and number (percentage). ^a^ the comparison between No/Mild group and Moderate/Severe group; ^b^ the comparison between No/Mild group and Edentulous group; ^c^ the comparison between Moderate/Severe group and Edentulous group

Significant difference ^*^ P < 0.05, ^**^ P < 0.01, ^***^ P < 0.001

**Table 3:** Radiological data of included patients in No/Mild group, Moderate/Severe group, and edentulous group

	No/Mild group	Moderate/Severe group	Edentulous group
Pfirrmann grade of disc degeneration			
L1-L2, n (%)			
**No degeneration**	18 (27.7)	12 (21.1) ^c*^	9 (47.4)
**Degeneration**	47 (72.3)	45 (78.9)	10 (52.6)
**Moderate degeneration (IV)**	44 (67.7)	41 (71.9)	10 (52.6)
**Severe Degeneration (V)**	3 (4.6)	4 (7.0)	0
**L2-L3, n (%)**			
**No degeneration**	15 (23.1)	9 (15.8) ^c*^	8 (42.1)
**Degeneration**	50 (76.9)	48 (84.2)	11 (57.9)
**Moderate degeneration (IV)**	44 (67.7)	38 (66.7)	10 (52.6)
**Severe Degeneration (V)**	6 (9.2)	10 (17.5)	1 (5.3)
**L3-L4, n (%)**			
**No degeneration**	15 (23.1)	9 (15.8) ^c*^	8 (42.1)
**Degeneration**	50 (76.9)	48 (84.2)	11 (57.9)
**Moderate degeneration (IV)**	42 (64.6)	44 (77.2)	10 (52.6)
**Severe Degeneration (V)**	8 (12.3)	4 (7.0)	1 (5.3)
**L4-L5, n (%)**			
**No degeneration**	6 (9.2) ^b*^	6 (10.5) ^c*^	6 (31.6)
**Degeneration**	59 (90.8)	51 (89.5)	13 (68.4)
**Moderate degeneration (IV)**	47 (72.3)	43 (75.4) ^c*^	7 (36.8)
**Severe Degeneration (V)**	12 (18.5)	8 (14.1)	6 (31.6)
**L5S1, n (%)**			
**No degeneration**	8 (12.3) ^b*^	6 (10.5) ^c*^	6 (31.6)
**Degeneration**	57 (87.7)	51 (89.5)	13 (68.4)
**Moderate degeneration (IV)**	32 (49.2)	34 (59.6)	8 (42.1)
**Severe Degeneration (V)**	25 (38.5)	17 (29.8)	5 (26.3)
**Endplate change**			
**L1 (yes), n (%)**	2 (3.1)	0	1 (5.3)
**L2 (yes), n (%)**	9 (13.8)	4 (7.0)	1 (5.3)
**L3 (yes), n (%)**	12 (18.5)	6 (10.5)	2 (10.5)
**L4 (yes), n (%)**	19 (29.2) ^b*^	16 (28.1) ^c*^	1 (5.3)
**L5 (yes), n (%)**	19 (29.2) ^b*^	16 (28.1) ^c*^	1 (5.3)
**S1 (yes), n (%)**	10 (15.4)	6 (10.5)	2 (10.5)
**ROM (°)**			
**L1-L2**	2.56 ± 1.39	2.94 ± 2.52	3.01 ± 1.98
**L2-L3**	3.51 ± 2.05	3.17 ± 2.47	3.18 ± 2.05
**L3-L4**	4.22 ± 2.84	3.69 ± 2.58	3.41 ± 2.58
**L4-L5**	4.25 ± 2.68	4.81 ± 3.36	5.03 ± 3.15
**L5S1**	5.30 ± 2.82	4.44 ± 2.22	5.49 ± 3.31
**LL**	31.20 ± 12.69	30.36 ± 15.20	31.23 ± 11.00
**PI**	50.75 ± 11.66	51.89 ± 9.36	50.68 ± 11.06
**PT**	24.08 ± 10.58	24.69 ± 8.03	27.37 ± 11.94
**SS**	26.69 ± 7.76	27.25 ± 9.01	23.29 ± 8.94
**Disc height (mm)**			
**L1-L2**			
**Anterior**	9.76 ± 2.17	9.78 ± 2.85	10.23 ± 2.47
**Middle**	9.72 ± 2.16	9.56 ± 2.78	10.20 ± 2.26
**Posterior**	6.62 ± 1.63	6.94 ± 1.72	6.61 ± 1.88
**L2-L3**			
**Anterior**	10.95 ± 2.72	10.34 ± 3.54	10.81 ± 2.66
**Middle**	10.30 ± 3.54	9.74 ± 3.27	9.82 ± 2.70
**Posterior**	7.22 ± 1.86	7.11 ± 2.10	7.53 ± 2.30
**L3-L4**			
**Anterior**	12.47 ± 3.72	12.18 ± 4.19	12.13 ± 3.56
**Middle**	10.78 ± 3.16	10.85 ± 3.53	10.85 ± 3.11
**Posterior**	7.45 ± 2.00	8.33 ± 2.45	8.43 ± 2.02
**L4-L5**			
**Anterior**	12.33 ± 3.80	13.09 ± 3.89	12.16 ± 2.88
**Middle**	10.56 ± 3.21	11.24 ± 3.55	10.46 ± 2.45
**Posterior**	8.36 ± 2.00	8.63 ± 2.85	8.29 ± 1.85
**L5S1**			
**Anterior**	13.46 ± 4.41	14.72 ± 3.78	14.57 ± 4.44
**Middle**	10.06 ± 3.33	11.41 ± 3.75	11.77 ± 3.17
**Posterior**	8.28 ± 2.15	8.70 ± 2.42	8.68 ± 1.49

LL - lumbar lordosis; PI - pelvic incidence; PT - pelvic tilt; SS - sacral slope, ROM – range of motion; Continuous and dichotomous data are presented as mean ± standard deviation and number (percentage)

^a^ the comparison between No/Mild group and Moderate/Severe group; ^b^ the comparison between No/Mild group and Edentulous group; ^c^ the comparison between Moderate/Severe group and Edentulous group

Significant difference ^*^ P < 0.05, ^**^ P < 0.01

#### No/Mild group versus edentulous group

The VAS LBP (P = 0.025) and VAS leg pain (P = 0.008) of patients preoperatively in the No/Mild group were significantly higher compared with the edentulous group (Table 2). The incidence rate of IVD degeneration in L3-L4, L4-L5, and L5S1 based on Pfirrmann grade and endplate changes in the fourth and fifth lumbar vertebrae in the No/Mild group were significantly higher compared with the edentulous group (Tables 2 and 3).

#### Moderate/Severe group versus edentulous group

The VAS LBP (P = 0.027) and VAS leg pain (P = 0.018) of patients preoperatively in the Moderate/Severe group were significantly higher compared with the edentulous group (Table 2). The incidence rate of IVD degeneration in L1-L2, L2-L3, L4-L5, and L5S1 based on Pfirrmann grade and endplate changes in the fourth and fifth lumbar vertebrae in the Moderate/Severe group were significantly higher compared with the edentulous group (Tables 2 and 3).

#### Association between Periodontal Parameters and clinical outcomes

There was a positive association between PLI and pain scores (VAS LBP: r = 0.215, P = 0.030, and VAS leg pain: r = 0.309, P = 0.005), but no significant difference in ODI score. The number of remaining teeth, BI, GR, and PD were not related to pre-operative clinical outcomes (including VAS LBP, VAS leg pain, and ODI) (Table 4).

**Table 4:** Associations between periodontal parameters and radiological changes, and between periodontal parameters and clinical outcomes

	Plaque index (PLI)	Bleeding index (BI)	Gingival recession (GR)	Probing depth (PD)	Number of remaining teeth
**VAS LBP**	0.215 (0.030) ^*^	-0.100 (0.317)	0.170 (0.088)	-0.080 (0.428)	-0.143 (0.151)
**VAS leg pain**	0.309 (0.005) ^**^	-0058 (0.608)	0.112 (0.321)	-0.013 (0.911)	-0.199 (0.075)
**ODI**	0.052 (0.691)	0.080 (0.539)	0.077 (0.553)	-0.102 (0.439)	-0.074 (0.570)
**ROM (°)**					
**L1-L2**	0.078 (0.424)	0.042 (0.669)	0.170 (0.081)	-0.012 (0.905)	-0133 (0.173)
**L2-L3**	0.140 (0.151)	0.080 (0.415)	0.065 (0.506)	0.051 (0.606)	0.036 (0.712)
**L3-L4**	0.092 (0.345)	0.088 (0.367)	-0.045 (0.649)	0.049 (0.617)	-0.032 (0.740)
**L4-L5**	-0.022 (0.822)	-0.059 (0.546)	0.123 (0.208)	0.004 (0.965)	-0.014 (0.884)
**L5S1**	0.011 (0.914)	0.056 (0.566)	0.087 (0.375)	0.002 (0.982)	0.048 (0.625)
**LL**	0.072 (0.435)	0.005 (0.956)	-0.029 (0.752)	0.014 (0.877)	0.112 (0.223)
**PI**	0.096 (0.292)	0.026 (0.776)	0.017 (0.851)	-0.038 (0.681)	0.039 (0.668)
**PT**	0.071 (0.437)	0.034 (0.712)	0.047 (0.609)	-0.057 (0.537)	-0.056 (0.545)
**SS**	0.041 (0.652)	-0.012 (0.892)	-0.032 (0.725)	0.012 (0.898)	0.113 (0.217)
**Disc height (mm)**					
**L1-L2**					
**Anterior**	0.086 (0.351)	0.059 (0.523)	0.113 (0.216)	0.149 (0.105)	0.005 (0.959)
**Middle**	0.137 (0.134)	0.055 (0.545)	0.125 (0.172)	0.153 (0.095)	-0.113 (0.217)
**Posterior**	0.061 (0.506)	0.031 (0.738)	0.096 (0.294)	0.108 (0.239)	-0.046 (0.616)
**L2-L3**					
**Anterior**	-0.144 (0.116)	-0.084 (0.362)	-0.171 (0.061)	-0.035 (0.708)	0.121 (0.186)
**Middle**	0.015 (0.870)	0.034 (0.715)	-0.035 (0.701)	0.084 (0.363)	-0.075 (0.416)
**Posterior**	-0.049 (0.596)	-0.110 (0.229)	-0.037 (0.685)	-0.057 (0.537)	0.001 (0.995)
**L3-L4**					
**Anterior**	-0.016 (0.862)	-0.056 (0.543)	0.131 (0.151)	-0.017 (0.852)	-0.115 (0.209)
**Middle**	0.009 (0.925)	0.006 (0.946)	0.120 (0.189)	0.040 (0.662)	-0.091 (0.319)
**Posterior**	0.033 (0.718)	0.043 (0.642)	0.101 (0.253)	0.121 (0.189)	-0.052 (0.569)
**L4-L5**					
**Anterior**	0.054 (0.553)	0.075 (0.412)	0.165 (0.071)	0.139 (0.131)	-0.098 (0.283)
**Middle**	0.038 (0.678)	0.172 (0.059)	0.121 (0.188)	0.175 (0.056)	-0.049 (0.593)
**Posterior**	-0.019 (0.837)	0103 (0.261)	0.019 (0.834)	0.056 (0.542)	0.008 (0.929)
**L5S1**					
**Anterior**	0.051 (0.576)	0.134 (0.143)	0.114 (0.214)	0.136 (0.139)	0.074 (0.418)
**Middle**	0.102 (0.266)	0.108 (0.222)	0.108 (0.240)	0.175 (0.056)	0.032 (0.726)
**Posterior**	0.122 (0.184)	0.129 (0.159)	0.126 (0.115)	0.165 (0.072)	-0.072 (0.433)

BMI - body mass index, VAS - visual analogue scale, LBP - low back pain, ODI – Oswestry disability index, LL - lumbar lordosis; PI - pelvic incidence; PT - pelvic tilt; SS - sacral slope, ROM – range of motion. Data was presented as coefficient value (P value)

Significant difference ^*^ P < 0.05, ^**^ P < 0.01 (Pearson’s correlation coefficient)

#### Association between Periodontal Parameters and Radiological Data

There was no significant correlation between the periodontal parameters (including number of remaining teeth, PLI, BI, GR, and PD) and radiological outcomes (including ROM of each lumbar segmental level, LL, PI, PT, SS, and disc height of each lumbar segmental level) (Table 4).

#### Inter-rater reliability

There was good to excellent agreement in terms of inter-rater for the radiological data (including ROM: 0.086 (0.855, 0.911), disc height: 0.842 (0.833, 0.891), LL: 0.844 (0.831, 0.887), PI: 0.801 (0.788, 0.829), PT: 0.902 (0.884, 0.9177), AND SS; 0.864 (0.846, 0.904)).

## Discussion

To the best of our knowledge, the results of this study provide the first evidence of the relationships between periodontitis severity and disc degeneration and endplate change in older adults with lumbar degenerative disorders. We found out that periodontal clinical parameters can differ between dentate and edentulous groups. Data show a higher incidence rate of IVD degeneration and endplate change in some specific level(s) in individuals with periodontitis. The PLI is positively associated with VAS LBP and leg pain. These findings have potential implications for understanding the mechanisms of underlying the mouth-gut-disc axis.

### Associations between periodontitis and IVD degeneration and endplate change

**Fig. 1:**
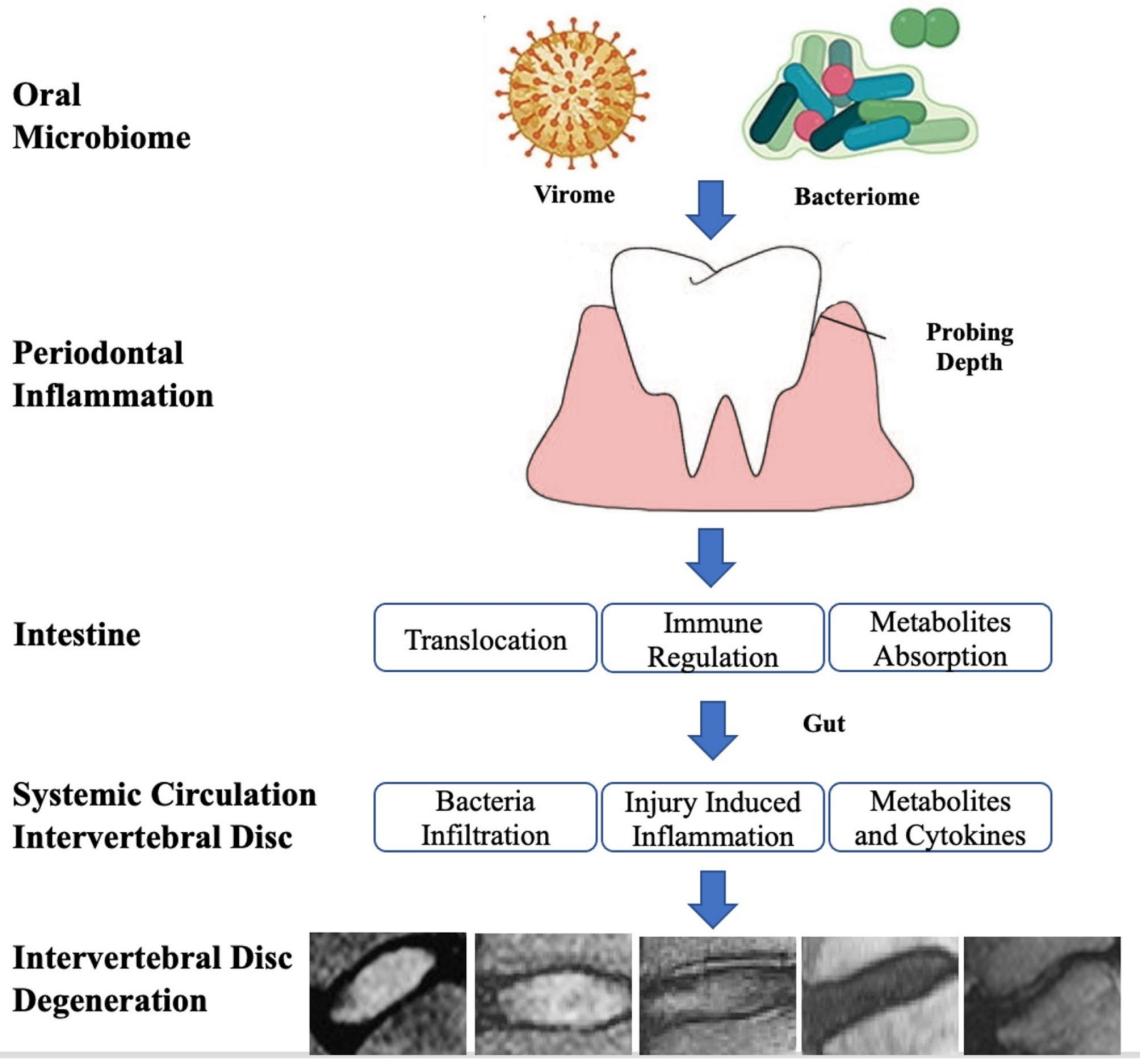
Mouth-gut-axis. Oral microorganisms (e.g., virus and bacteria) form a complex ecosystem that thrive in the dynamic oral environment. The microbial composition is significantly affected by interspecies and host-microbial interactions, which in turn, as the main cause for the occurrence of periodontitis. The microorganisms and related inflammatory cytokines can be delivered into the intestine system. Gut microbes alter the intestinal microbial environment by modulating gut microbiota translocation and composition, increasing intestinal permeability and inflammation and modifying metabolites absorption. Related responses from these changes have been delivered into the intervertebral disc via systemic circulation system which can cause intervertebral disc degeneration [11]

In the present study, the higher periodontitis parameters (PLI, BI, GR, and PD), the incidence rate of IVD degeneration in all lumbar spine segments and endplate change in the fourth and fifth lumbar vertebrae in the dentate group could be potentially explained by the loop of mouth-gut-disc axis due to the microbiome composition (Fig. 1).

### Association between periodontitis and microbiome

A previous study showed that more than 600 bacterial species have been detected in the periodontal pockets from the progressive destruction of the gum, periodontal membrane, and alveolar bone in patients with periodontitis [25, 26]. The human oral microbiome is one of the most frequently studies human microflora that interacts with the mucosal immune system through a balanced equilibrium between symbiotic or pathogenic factors and the defense mechanisms of the immune system [26, 27]. Intriguingly, the results from a meta-analysis showed that alterations in the microbiome composition rather than single targeted pathogens in the mouth are found to be associated with an increased risk for the development of periodontitis [13].

### Gut-disc axis

A recent, comparable study showed that the composition of the microbiome in patients with healthy IVD differed from those with degenerative IVD and herniated IVD [28]. This study also showed the presence of 58 overlapping bacterial species between the IVDs and the gut, and 29 overlapping bacterial species between the IVDs and the skin. This bacterial picture suggests that the IVD microbiome may have an interplay with the gut microbiome; the hypothesis is that the gut microbiome infiltrates the IVD environment and plays a key role in the development of IDD. The theory that microbiome dysbiosis may be an important cause of inflammation and IDD requires future validation in adequately powered, prospective registries.

A recent review listed three potential mechanisms for the establishment of the gut-disc axis (11): (1) The delivery of bacteria through the gut epithelial barrier into IVDs;(2) Bacterial regulatory action of the mucosal and systemic immune system;(3) Regulation of nutrient absorption and metabolite formation at the gut epithelium level.

Although IVD is multifactorial, low-virulence anaerobic bacteria may be a cofactor in uncontrolled low-grade inflammation in IVD [29, 30]. The mechano-immunological and infectious pathways that lead to IVD all theoretically accelerate tissue damage in the disc. Previous published systematic reviews and observational studies suggest a significantly higher prevalence of bacterial infection in patients with disc disease or degeneration [29–32]. Furthermore, there is growing evidence to support the presence of inflammation in association with mechanical insults as a contributor to the development of endplate changes [33]. At present some studies have demonstrated a significant association between low virulence anaerobic bacteria and pathogenesis of endplate change [34–36]. Of note, previous studies supported the associations between the microbiome composition in the gastrointestinal system and mouth with a variety of chronic diseases, including autoimmune disease, gut inflammation disorders, cardiometabolic diseases, chronic kidney disease, neurological and respiratory diseases, mental health disorders, and osteoarthritis [37–42]. Although oral microorganisms or gut microbiota have been considered as the main potential trigger, the source of the low virulence anaerobic bacteria for the pathogenesis of IVD and endplate change is still unclear. The mechanisms of the mouth-gut-disc axis should be interpreted with prospective randomized controlled studies with a large number of participants which are warranted to investigate the source and role of microbiome dysbiosis in the pathogenesis of symptomatic IVD degeneration and endplate change.

### The affection of edentulous and periodontitis severity

In our study, the dentate participants with a higher incidence rate of disc degeneration and endplate change and higher VAS LBP and leg pain could be potentially explained by the changing of oral microbiota in dentate and edentulous groups. The study shows that periodontal pathogens (e.g., anaerobic species) live in distinct sites in the oral cavity. The complete loss of teeth in the edentulous group causes a breakdown in this microbial habitat, leading to alterations in the oral microbiome composition [43, 44]. Notably, the mouth-gut-disc axis could potentially result in disc degeneration and endplate change.

The previous study showed that there are significant differences in the microbial composition between severe and mild periodontitis in the subgingival microbiome (i.e., pocket samples) and this is positively associated with systemic inflammatory markers [14]. Systemic inflammation in severe periodontitis may be driven by the oral microbiome and may support the indirect (inflammatory) mechanism for the higher incidence rate of IVD degeneration and endplate change in some specific level(s) in individuals with severe periodontitis in our study.

### Association between periodontitis and clinical outcome

In theory, the severity of PLI is associated with the alterations in oral microbiome composition and host responses to the microbiota, resulting in the severity of periodontitis, which may cause pathological bone development and involution and systemic inflammatory response. Moreover, the local and systemic inflammatory responses may lead to IVD degeneration and/or endplate change and related pain. Furthermore, bacterial invasion into the IVDs dysregulates the local and/or systemic inflammatory response, which stimulates the secretion of inflammatory cytokines, and induces pro-inflammatory phenotypes of immune cells. Due to increased innervation of the degenerative IVDs, these cascade responses lead to pain amplification and the transmission of pain signals to peripheral afferent nerve fibers located in the dorsal root ganglia (DRG) and brain [45].

Although we did not find a linear correlation between oral microbiome dysbiosis, LBP, and disability, we think that this may reflect a major limitation of our study. The studies we captured omitted patients who did not have clinical data on back pain available, therefore reducing our available sample size for analysis.

### Methodological issues

Several methodological issues required consideration. First, there is a lack of direct evidence of oral microbial pathogens in lumbar IVD degeneration and endplate change. Second, healthy participants as the control group is missing. Third, the systemic and local inflammatory responses for the microbiome dysbiosis in periodontitis are missing. Fourth, the history of antibiotic use is missing. Finally, the tissue samples of lumbar IVD haven’t been tested for etiological detection, pathological examination, and laboratory test for analyzing the sequence of all protein-coding nuclear genes in the genome. A future prospective study should investigate the direct correlation between oral microbial pathogens and IVD degeneration/endplate change and clinical outcomes using a randomized controlled design with a larger sample size.

## Conclusion

The novel results presented here support the hypothesis that the severity of periodontitis is associated with the higher incidence rate of IVD degeneration and endplate change and clinical outcomes in older adults with lumbar degenerative disorders. Furthermore, the discovery of these relationships unveils a novel mechanism through which the alterations in oral microbiome composition potentially promote IVD degeneration and pain. They provide new insights into the oral pathogens in lumbar degenerative disorders with LBP and provide initial indirect evidence for the translation of some, but not all, observations from recent animal studies to humans.

## Data Availability

The data that support the findings of this study are available from the corresponding author, Xiaolong Chen, upon reasonable request.
